# Chinese Massage Combined with Herbal Ointment for Athletes with Nonspecific Low Back Pain: A Randomized Controlled Trial

**DOI:** 10.1155/2012/695726

**Published:** 2012-11-13

**Authors:** Ling Jun Kong, Min Fang, Hong Sheng Zhan, Wei An Yuan, Ji Ming Tao, Gao Wei Qi, Ying Wu Cheng

**Affiliations:** ^1^Yueyang Hospital of Integrated Traditional Chinese and Western Medicine, Shanghai University of Traditional Chinese Medicine, Shanghai 200437, China; ^2^Research Institute of Tuina, Shanghai Academy of Traditional Chinese Medicine, Shanghai 201203, China; ^3^Department of Orthopedics, Shuguang Hospital, Shanghai University of Traditional Chinese Medicine, Shanghai 201203, China; ^4^Department of Traditional Chinese Medicine, Songjiang People Hospital of Shanghai, Shanghai 201699, China

## Abstract

Non-specific low back pain (NLBP) is an increasing health problem for athletes. This randomized controlled trial was designed to investigate the effects of Chinese massage combined with herbal ointment for NLBP. 110 athletes with NLBP were randomly assigned to experimental group with Chinese massage combined with herbal ointment or control group with simple massage therapy. The primary outcome was pain by Chinese Short Form McGill Pain Questionnaire (C-SFMPQ). The secondary outcome was local muscle stiffness by Myotonometer. After 4 weeks, the experimental group experienced significant improvements in C-SFMPQ and in local muscle stiffness compared with control group (between-group difference in mean change from baseline, −1.24 points, *P* = 0.005 in sensory scores; −3.14 points, *P* < 0.001 in affective scores; −4.39 points, *P* < 0.001 in total scores; −0.64 points, *P* = 0.002 in VAS; −1.04 points, *P* = 0.005 in local muscle stiffness during relaxation state). The difference remained at one month followup, but it was only significant in affective scores (−2.83 points, *P* < 0.001) at three months followup. No adverse events were observed. These findings suggest that Chinese massage combined with herbal ointment may be a beneficial complementary and alternative therapy for athletes with NLBP.

## 1. Introduction

Chinese massage combined with herbal ointment is one of the centuried complementary and alternative therapies for improving pain, anxiety, muscle stiffness, and so forth. In China, the essential oils were extracted from traditional Chinese herbs by steam distillation or infusions [1]. The Chinese herbs were chosen due to the known analgesic, anti-inflammatory, antispasmodic, and carminative effects [19]. In order to use and carry expediently, the essential oils from the Chinese herbs are usually mixed in vaseline, glycerine, lard, and so forth [2].

The incidence of nonspecific low back pain is increasing in athletes due to participating in a number of sports compared with age-related peers [3, 4]. And Chinese massage combined with herbal ointment is one of the most popular complementary and alternative therapies for athletes with nonspecific low back pain in China. However, the trials were relatively few, which studied the effect of Chinese massage combined with herbal ointment for symptom improvements in athletes with nonspecific low back pain. Although some studies showed short-term effect of this alternative therapy for pain and local muscle stiffness due to nonspecific low back pain [5–8], the methodological quality of these studies were poor.

The objective of this randomized controlled trial was to determine whether a course of Chinese massage combined with herbal ointment confers greater improvement on pain due to nonspecific low back pain than simple massage therapy in athletes. In addition, we also examined whether this alternative therapy produced greater improvement on local muscle stiffness caused by nonspecific low back pain. And the followup of one and three months were also performed.

## 2. Materials and Methods

### 2.1. Design

We used a pragmatic randomized controlled trial to evaluate the effectiveness on Chinese massage combined with herbal ointment for athletes with nonspecific low back pain. Patients were randomly assigned to experimental group receiving Chinese massage combined with herbal ointment or control group experiencing simple massage therapy by computer generated numbers. The randomized treatment assignments were sealed in opaque envelopes and opened after the baseline assessment. The patients in two groups received two 30-min interventions weekly for 4 weeks. And in order to blind patients and therapists, the placebo ointment was applied in control group.

### 2.2. Preparation of the Herbal Ointment

First, preparation of the cream base: white vaseline, stearic acid, cetyl alcohol, lanolin, and propylene paraben were mixed and heated to the fusion point as the oil phase. The mixture of glycerin, polysorbate, emulsifier op-10, and deionized water was heated as the aqueous phase. These two separate phases were mixed continuously while being cooled as the cream base. Second, extracting essential oils: equal powders of Dang Gui (*Radix Angelicae Sinensis*), Chuan Xiong (*Rhizoma chuanxiong*), Xi Xin (*Radix et Rhizoma Asari*), and Rou Gui (*Cortex Cinnamomi*) were immersed in water (powder/water proportion: 1 : 10) for 2 hours. And then the essential oils were extracted from this mixed liquor by steam distillation. Last, the essential oils were added to the cream base as the finished product of the herbal ointment (20 g essential oils per each 100 g herbal ointments). The entire process was carried out under sterile conditions. The cream base was used as the placebo ointment in control group.

### 2.3. Subjects

Patients were recruited from athletes with nonspecific low back pain in Shanghai Institute of Physical Education and Shanghai Sports Center of Shooting and Archery between January 2008 and October 2009. 

Inclusion criteria: (1) aging 15–35 years; (2) having nonspecific low back pain without any relevant ongoing pathologies such as disc prolapse, fractures, spondylolisthesis, tumor, osteoporosis, or infection; (3) willing to participate in this study; signing informed consent.

Exclusion criteria: (1) having other pain syndromes; (2) experiencing spinal surgery in the past 6 months or having to undergo surgery or invasive examinations during the study; (3) having neurological disease; (4) having psychiatric disease; (5) having serious chronic disease that could interfere with the outcomes (e.g., cardiovascular disease, rheumatoid arthritis, epilepsy, or other disqualifying conditions); (6) pregnant or planning to become pregnant during the study; (7) failing to communicate in Chinese.

### 2.4. Treatment

In experimental group, the patients received Chinese massage including palm friction, stroking, petrissage, rolling, and tapotement; it was performed in the low back for 30 min by professional therapists after the applying above herbal ointment. In control group, patients experienced the same treatment protocol of Chinese massage after applying the placebo ointment in the low back. 

### 2.5. Measurements

All outcomes were assessed by observers unaware of the grouping, at baseline (M1), immediately after the first intervention (M2), and immediately after the last intervention (M3). The followup included the assessments at one month (M4) and three months (M5) after the last intervention. 

The primary outcome measure was the change in pain by the Chinese Short Form McGill Pain Questionnaire (C-SFMPQ). The main component of the C-SFMPQ consists of 15 descriptors (11 sensory including throbbing, shooting, stabbing, sharp, cramping, gnawing, hot burning, aching, heavy, tender, and splitting; 4 affective including tiring-exhausting, sickening, fearful, and punishing cruel), which are rated on an intensity scale as 0 = none, 1 = mild, 2 = moderate, or 3 = severe. Three pain scores are derived from the sum of the intensity rank values of the words chosen for sensory, affective, and total descriptors. The C-SFMPQ also includes a visual analogue scale (VAS, rang 0 to 10, with higher scores indicating greater pain) [9].

The secondary outcome measure was the change in local muscle stiffness by Myotonometer (Neurogenic Technologies Inc, Missoula, MT, USA). For all measurements, the patients took a prone position in the bed. The Myotonometer probe was located halfway of the belly of lumbar erector spine muscle. The probe consists of an outer cylinder that remains stationary as an inner cylinder pushes onto and compresses the underlying tissue. The distance between the outer and inner cylinders determines tissue displacement. The inner cylinder houses a force transducer that measures the amount of tissue resistance as the probe compresses the underlying tissue. Eight displacement measurements, corresponding to 8 increments of force (0.25, 0.50, 0.75, 1.00, 1.25, 1.50, 1.75, and 2.00 kg), are obtained. Computational software creates force-displacement curves based on these data. A more compliant (lower stiffness) muscle will have more displacement per unit force than a muscle with less compliance (higher stiffness) [10, 11]. So the area under the curves is indicative of the level of severity of the muscle stiffness condition. 

The muscle was tested during a relaxed state (RS) and a maximal voluntary contraction (MVC). The assessor performed 5 probes on the pain side of the low back during RS. Computational software automatically averaged these measurements into a single data point for each force increment (0.25 to 2.00 kg) and calculated the area under this averaged curves. The same procedure was performed during MVC. There was a 30-second rest period between measurements.

### 2.6. Statistical Analysis

Our pretrial power calculation indicated that 86 patients (50% to each group) were required to detect a difference in pain relief at a significance level of 5% (a two-sided *t*-test) with 80% power. In anticipation of a 20% attrition rate, we sought 108 patients at least.

Between-group difference at baseline was analyzed using Independent-samples *t*-test or Chi-square test. Changes in continuous measures were analyzed by analysis of variance (ANOVA). Effects were evaluated on an intention-to-treat basis, and participants who did not complete the followup period were considered not to have had any changes in scores. A two-sided *P* value of less than 0.05 indicated statistical significance. Results are presented as mean and standard deviation (SD) at M1 and as between-group difference with 95% confidence intervals (CI) at M2, M3, M4, and M5.

## 3. Results

**Figure 1:**
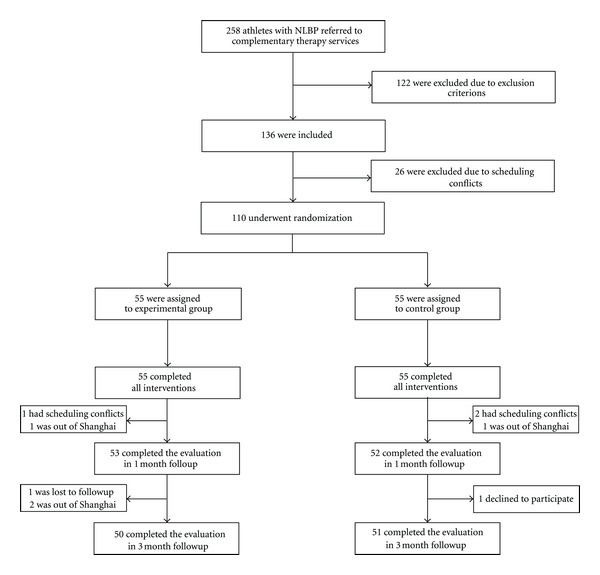
Screening, randomization, and completion evaluations from the baseline to three months followup, NLBP = nonspecific low back pain.

Between January 2008 and October 2009, 258 athletes chose complementary therapies for nonspecific low back pain. 122 were rejected due to exclusion criterions. And then 26 patients could not participate in random assignment, because they had scheduling conflicts. Thus, 110 eligible participants were randomly assigned in equal number to either experimental group or the control group. All participants completed the 4-week interventions. After 1 month or 3 months, the rate of attendance was 96% or 91% for experimental group, and 95% or 93% for control group (Figure 1).

### 3.1. Baseline Characteristics of the Patients

Table 1 shows the baseline data for the 110 participants. Athletes from shooting, archery, and handball had a mean age of 21 years, and 48% were women. The rate of chronic pain due to nonspecific low back pain was 49% for experimental group, and 55% for control group. Although most patients took analgesics before interventions and some took anticonvulsants, the two groups were reasonably balanced. And the baseline outcome including C-SFMPQ, VAS, and muscle stiffness scores were also reasonably well balanced between experimental group and control group.

### 3.2. Improvement in the Primary Outcome

**Figure 2:**
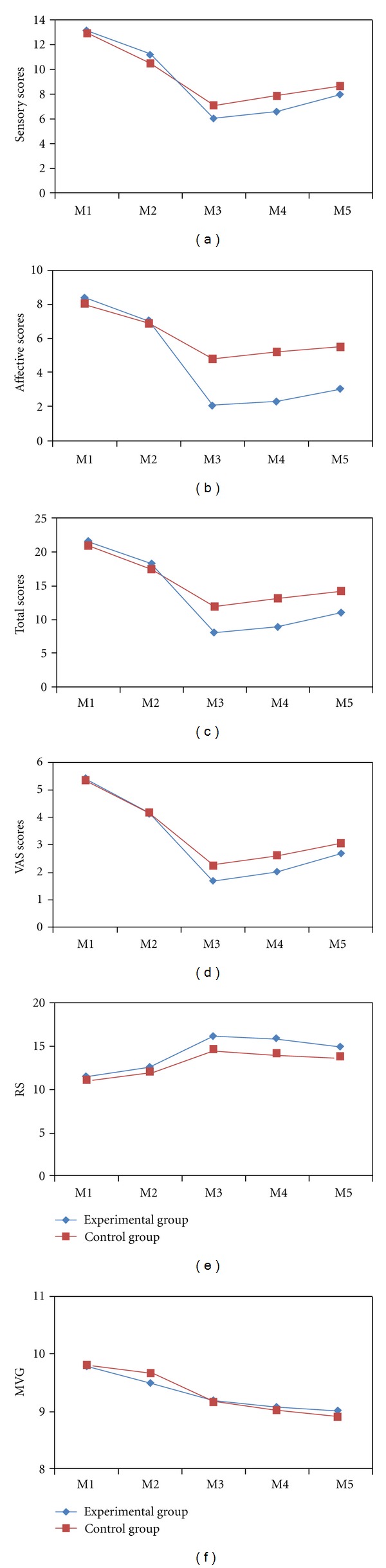
Mean changes of the primary and secondary outcomes. The means of outcomes are shown for the experimental group (triangles) and the control group (squares). Measurements were obtained at baseline (M1), immediately after the first intervention (M2), immediately after the last intervention (M3), one month (M4), and three months (M5) after the last intervention. The Chinese Short Form McGill Pain Questionnaire (C-SFMPQ) consists of 15 descriptors (11 sensory; 4 affective) which are rated on an intensity scale (0 to 3), such the C-SFMPQ scores consist of sensory scores, affective scores, and total scores with the higher scores indicating greater pain. Visual analogue scale (VAS, rang 0 to 10) with higher scores indicating greater pain. The local muscle stiffness was tested during a relaxed state (RS) or a maximal voluntary contraction (MVC) by Myotonometer with higher scores indicating lower stiffness.

The changes in the primary outcomes from baseline to three months followup are shown in Table 2 and Figure 2. Immediately after the first intervention, two groups showed greater decrease in C-SFMPQ (including sensory scores, affective scores, total scores, and VAS scores) than the baseline. But, between-group difference was not significant.

Immediately after the last intervention, two groups also had significantly greater reduction in C-SFMPQ than the baseline. And the mean between-group difference in the change from baseline to the end of the last intervention was significant in sensory scores (−1.24 points [95% confidence interval {CI}, −2.09 to −0.39]; *P* = 0.005), affective scores (−3.14 points [95% CI, −3.67 to −2.61]; *P* < 0.001), total scores (−4.39 points [95% CI, −5.61 to −3.17]; *P* < 0.001), and VAS scores (−0.64 points [95% CI, −1.04 to −0.24]; *P* = 0.002).

Improvements in two groups were maintained at one month after the last intervention for sensory scores, affective scores, total scores, and VAS scores. And the between-group difference also was significant in sensory scores (−1.46 points [95% CI, −2.41 to −0.51]; *P* = 0.003), affective scores (−3.29 points [95% CI, −3.84 to −2.74]; *P* < 0.001), total scores (−4.75 points [95% CI, −6.09 to −3.41]; *P* < 0.001), and VAS scores (−0.66 points [95% CI, −1.13 to −0.19]; *P* = 0.007). The changes from baseline to 6 months followup in C-SFMPQ remained significant in the experimental group and control group, but the between-group difference was only significant in affective scores (−2.83 points [95% CI, −3.54 to −2.12]; *P* < 0.001) and total scores (−3.71 points [95% CI, −5.48 to −1.94]; *P* < 0.001).

### 3.3. Improvement in the Secondary Outcome

Immediately after the first intervention, two groups had significant improvement in local muscle stiffness in a relaxation state than the baseline. But, between-group difference was not significant (−0.09 points [95% CI, −0.46 to 0.28]; *P* = 0.632). The between-group difference was significant (−1.04 points [95% CI, −1.76 to −0.32]; *P* = 0.005) at the end of the last intervention, and remained significant (−1.29 points [95% CI, −2.03 to −0.55]; *P* = 0.001) after one month followup, but not (−0.66 points [95% CI, −1.48 to 0.16]; *P* = 0.117) after three months followup.

In a maximal voluntary contraction, the local muscle stiffness did not showed significant improvement than the baseline immediately after the first intervention. The local muscle stiffness of two groups had significantly greater reduction after the last intervention, but the between-group difference was not significant until the end of three months followup. And the mean changes in a relaxation state and a maximal voluntary contraction were showed in Figure 2. 

### 3.4. Adverse Events

No adverse events were noted during the study interventions in either experimental group or control group. 

## 4. Discussion

Chinese massage combined with herbal ointment, as one of the complementary and alternative therapies, has a long history in China. It is similar with the aromatherapy massage. Two alternative therapies are manual therapy in combination with the topical applications of the essential oils. But, Chinese massage combined with herbal ointment had some characteristics compared with other aromatherapy massage. First, its essential oils were extracted from different Chinese herbs according to various aims (e.g., pain relief, improvement of anxiety, etc.). Second, in order to use and carry expediently, these essential oils from Chinese herbs were mixed in lard, vaseline, glycerine, and so forth. And the infusion by liquor is also chosen for improving the effects of various Chinese herbs [2]. In addition, Chinese massage combined with herbal ointment is popular for various diseases including neck pain, low back pain, dysmenorrhea, pediatric torticollis, and cibophobia in China [12–14].

This randomized controlled trial has shown that Chinese massage combined with herbal ointment was more effective for patients with nonspecific low back pain than simple massage therapy. The effect was evident in C-SFMPQ scores, a well-validated, multidimensional instrument for the assessment of pain including sensory scores, affective scores, and VAS scores after the last intervention. These benefits were sustained at the end of one month followup, and the benefit was still obvious in the affective scores at the end of three months followup. Although the between-group difference in the total scores was significant, it mainly was due to affective scores at the end of three months followup. The change of local muscle stiffness in a relaxation state was consistent with C-SFMPQ assessments. No adverse events were reported in the study participants, indicating that Chinese massage combined with herbal ointment might be a safe therapy for nonspecific low back pain.

Our results are consistent with previous, nonrandomized trials of Chinese massage combined with herbal ointment for nonspecific low back pain in China, especially in pain relief [5–7]. Our findings are also similar with observations from other clinical trials and reviews that support the benefits of aromatherapy for affective symptoms (e.g., anxiety, depression, etc.) management due to various diseases [15–18]. The benefits of Chinese massage combined with herbal ointment in affective scores of C-SFMPQ were sustained up to the end of three months followup. Aromatherapy massage is not commonly used for nonspecific low back pain in other countries, but Chinese massage combined with herbal ointment is popular for pain and some affective symptoms caused by nonspecific low back pain in China. 

The biologic mechanisms by which Chinese massage combined with herbal ointment might affect the clinical course of nonspecific low back pain remain unproven. But some possible basic mechanisms are popular for Chinese massage combined with herbal ointment or aromatherapy massage. Firstly, massage therapy promotes the pharmacological effect of the aromatic oils extracted from the Chinese herbs [19, 20]. Second, some studies supported that aromatherapy helps the body to improve immune response [21, 22]. In addition, some researchers maintains the aroma smell of most aromatic oils affects the nervous system through the olfactory system in aromatherapy massage [23, 24]. So the smell of the herbal ointment may have related therapeutic effects, although there are not related researches. 

Several limitations of the study are noted. First, the participants were athletes with nonspecific low back pain, which reduces the generalizability of the results. Second, in order to blind patients and therapists, the placebo ointment was applied in the control group. But the therapists found the difference between two groups because the smell of the Chinese herbs was difficult to mask. Although it might influence the trial, it does not mean that the blind method of the trial completely failed, because the impact has been limited. Blinding therapists can be difficult in this kind of trials. And there were no similar reports from the patients. In addition, the assessors of all outcomes were unaware of the grouping. Third, C-SFMPQ is a well-validated measurement tool for pain, but it only contains four affective descriptors including tiring-exhausting, sickening, fearful, and punishing cruel. It is insufficient to evaluate the affective changes compared with other measurement tools (e.g., Hospital Anxiety and Depression, HAD; Center for Epidemiologic Studies Depression, CES-D). Last but not the least, dysfunction is one of the major clinical symptoms caused by nonspecific low back pain. But our study did not evaluate the effect of Chinese massage combined with herbal ointment for the dysfunction due to nonspecific low back pain.

## 5. Conclusions

This randomized controlled trial makes a significant contribution to the body of evidence on the effectiveness of Chinese massage combined with herbal ointment in pain and local muscle stiffness due to nonspecific low back pain in athletes. Longer-term, double blinding, multicenter randomized controlled clinical trials are warranted to assess the generalizability of our findings and to deepen our understanding of this promising therapeutic approach.

## Figures and Tables

**Table 1:** Baseline characteristics of the study participants.*

Variable	Experimental group (*N* = 55)	Control group (*N* = 55)
Sex		
Male	29	28
Female	26	27
Age (years)	21.18 ± 3.77	19.95 ± 3.57
Sports: no. of patients		
Shooting	18	20
Archery	16	15
Handball	21	20
Duration of low back pain-related pain: no. of patients		
12 weeks or less	28	25
12 weeks or more	27	30
Medications before intervention: no. of patients (%)		
Analgesics	50 (91)	47 (85)
Anticonvulsants	15 (27)	18 (33)
C-SFMPQ scores^§^		
Sensory scores	13.13 ± 1.88	12.96 ± 1.86
Affective scores	8.38 ± 1.10	8.02 ± 1.08
Total scores	21.51 ± 2.54	20.98 ± 2.63
VAS scores^†^	5.42 ± 0.94	5.36 ± 1.04
Muscle stiffness scores^‡^		
Relaxed state	11.54 ± 1.42	10.98 ± 1.38
Maximal voluntary contraction	9.79 ± 0.92	9.50 ± 0.90

^∗^Plus-minus values are means ± SD unless otherwise noted.

^§^The Chinese Short Form McGill Pain Questionnaire (C-SFMPQ), which consists of 15 descriptors (11 sensory; 4 affective). Each descriptor is rated on an intensity scale (0 to 3) with the higher scores indicating greater pain.

^†^Visual analogue scale (VAS, rang 0 to 10) with higher scores indicating greater pain.

^‡^The local muscle stiffness was tested during a relaxed state or a maximal voluntary contraction by Myotonometer.

**Table 2:** Changes in primary and secondary outcomes.*

Variable	Mean change from baseline (95% CI)		Between-group difference (95% CI)	
	Experimental group (*N* = 55)	Control group (*N* = 55)	Experimental group versus control group	*P* value^§^
Sensory scores^†^				
M2	−1.91 (−2.64 to −1.18)	−2.43 (−3.09 to −1.77)	0.53 (−0.03 to 1.09)	0.070
M3	−7.11 (−7.87 to −6.35)	−5.87 (−6.72 to −5.02)	−1.24 (−2.09 to −0.39)	0.005
M4	−6.53 (−7.37 to −5.69)	−5.07 (−5.84 to −4.30)	−1.46 (−2.41 to −0.51)	0.003
M5	−5.17 (−6.09 to −4.25)	−4.29 (−5.18 to −3.40)	−0.88 (−2.11 to 0.35)	0.168
Affective scores^†^				
M2	−1.36 (−1.79 to −0.93)	−1.13 (−1.50 to −0.76)	−0.23 (−0.67 to 0.21)	0.290
M3	−6.34 (−6.68 to −6.00)	−3.20 (−3.74 to −2.66)	−3.14 (−3.67 to −2.61)	<0.001
M4	−6.11 (−6.48 to −5.74)	−2.82 (−3.34 to −2.30)	−3.29 (−3.84 to −2.74)	<0.001
M5	−5.36 (−5.82 to −4.90)	−2.53 (−3.10 to −1.96)	−2.83 (−3.54 to −2.12)	<0.001
Total scores^†^				
M2	−3.27 (−4.22 to −2.32)	−3.56 (−4.49 to −2.63)	0.29 (−0.60 to 1.18)	0.524
M3	−13.46 (−14.43 to −12.49)	−9.07 (−10.39 to −7.75)	−4.39 (−5.61 to −3.17)	<0.001
M4	−12.64 (−13.74 to −11.54)	−7.89 (−9.11 to −6.67)	−4.75 (−6.09 to −3.41)	<0.001
M5	−10.53 (−11.78 to −9.28)	−6.82 (−8.21 to −5.43)	−3.71 (−5.48 to −1.94)	<0.001
VAS scores^‡^				
M2	−1.26 (−1.60 to −0.92)	−1.18 (−1.55 to −0.81)	−0.07 (−0.36 to 0.22)	0.628
M3	−3.73 (−4.05 to −3.41)	−3.09 (−3.48 to −2.70)	−0.64 (−1.04 to −0.24)	0.002
M4	−3.40 (−3.77 to −3.03)	−2.74 (−3.13 to −2.35)	−0.66 (−1.13 to −0.19)	0.007
M5	−2.73 (−3.10 to −2.36)	−2.31 (−2.77 to −1.85)	−0.42 (−1.03 to 0.19)	0.181
RS^¶^				
M2	1.02 (0.53 to 1.51)	0.93 (0.43 to 1.43)	−0.09 (−0.46 to 0.28)	0.632
M3	4.62 (4.11 to 5.13)	3.58 (3.02 to 4.14)	−1.04 (−1.76 to −0.32)	0.005
M4	4.32 (3.81 to 4.83)	3.03 (2.46 to 3.60)	−1.29 (−2.03 to −0.55)	0.001
M5	3.36 (2.77 to 3.95)	2.70 (2.11 to 3.29)	−0.66 (−1.48 to 0.16)	0.117
MVC^¶^				
M2	−0.29 (−0.64 to 0.06)	−0.14 (−0.45 to 0.17)	0.15 (−0.08 to 0.38)	0.212
M3	−0.60 (−0.93 to −0.27)	−0.64 (−0.99 to −0.29)	−0.04 (−0.26 to 0.18)	0.700
M4	−0.71 (−1.04 to −0.38)	−0.79 (−1.11 to −0.47)	−0.08 (−0.33 to 0.17)	0.492
M5	−0.78 (−1.10 to −0.46)	−0.91 (−1.22 to −0.60)	−0.13 (−0.40 to 0.14)	0.328

^∗^All values are means with the 95% confidence intervals (CI). M2: immediately after the first intervention; M3: immediately after the last intervention; M4: one month after the last intervention; M5: three months after the last intervention.

^§^ *P* values were calculated with repeated measures analysis of variance.

^†^The Chinese Short Form McGill Pain Questionnaire (C-SFMPQ) consists of 15 descriptors (11 sensory; 4 affective). Each descriptor is rated on an intensity scale (0 to 3) with the higher scores indicating greater pain.

^‡^Visual analogue scale (VAS, rang 0 to 10) with higher scores indicating greater pain.

^¶^The local muscle stiffness was tested during a relaxed state (RS) or a maximal voluntary contraction (MVC) by Myotonometer with higher scores indicating smaller stiffness.
